# Comprehensive genomic profiling of a metastatic small cell lung carcinoma with a complete and long-term response to atezolizumab: A case report

**DOI:** 10.1016/j.rmcr.2023.101920

**Published:** 2023-09-26

**Authors:** Kresimir Tomic, Dragana Karan Krizanac, Faruk Skenderi, Kristina Krpina, Andrea Carapina Bilic, Kristina Galic, Zoran Gatalica, Semir Vranic

**Affiliations:** a Department of Oncology, University Clinical Hospital Mostar, Mostar, Bosnia and Herzegovina; b Department of Pathology, Cytology and Forensic Medicine, University Clinical Hospital Mostar, Mostar, Bosnia and Herzegovina; c Department of Pathology, UniMed Clinic, Sarajevo School of Science and Technology, Sarajevo, Bosnia and Herzegovina; d Clinic for Respiratory Diseases Jordanovac, University Hospital Centre Zagreb, Zagreb, Croatia; e Department of Family Medicine, Health Care Center Mostar, Mostar, Bosnia and Herzegovina; f Department for Lung Diseases, University Clinical Hospital Mostar, Mostar, Bosnia and Herzegovina; g Consultant Pathologist, Scottsdale, AZ, USA; h College of Medicine, QU Health, Qatar University, Doha, Qatar

**Keywords:** Lung cancer, Small cell lung cancer, Immunotherapy, Atezolizumab, Genomic profiling

## Abstract

Small cell lung cancer (SCLC) is a highly aggressive malignancy with a poor outcome. We present the case of a 57-year-old male patient with extensive-stage (ES-SCLC) treated with chemotherapy and atezolizumab. A complete response was achieved with a long remission of ∼three years. Comprehensive genomic profiling (CGP) of the tumor revealed high tumor mutation burden (13 mutations/Mb) and mutations of *TP53, RB1* and *ERCC4* genes. This case study confirms that a complete response to chemoimmunotherapy may be achieved in the case of ES-SCLC. It further provides the additional value of CGP and predictive testing in the management of ES-SCLC.

## 1 Introduction

Lung cancer is a leading cause of cancer morbidity and mortality worldwide [1]. SCLC accounts for 15% of all lung cancers and is marked by aggressive disease and poor outcomes [2]. Most patients have extensive-stage SCLC (ES-SCLC) with the spread of the disease outside the thorax due to highly proliferative activity. In the early localized stage of SCLC (LS-SCLC), a small percentage of patients can potentially be cured by chemoradiotherapy or surgery. In the ES-SCLC rapid relapse with distant metastases occur in most patients regardless of an excellent initial response to chemotherapy. The median survival of patients with ES-SCLC is 6–12 months, with currently available treatment options and rare long-term progression-free survival (PFS) [3].

The treatment of ES-SCLC has traditionally been a combination of platinum-based chemotherapy (cisplatin or carboplatin) and etoposide, with an overall response rate (partial or complete) of 60–70% [4]. Immunotherapy with immune checkpoint inhibitors (ICI) has revolutionized and transformed treatment in various malignancies, including ES-SCLC. Recently, a programmed death ligand 1 (PD-L1)-targeted ICI, atezolizumab or durvalumab immunotherapy, plus platinum with etoposide chemotherapy became the standard first-line treatment for ES-SCLC [5,6]. The approval of atezolizumab was based on the IMpower133 randomized clinical trial (RCT). It investigated the efficacy of atezolizumab or placebo IV in combination with carboplatin and etoposide for four 21-day cycles followed by maintenance atezolizumab or placebo until unacceptable toxicity, disease progression or no additional clinical benefit in previously untreated patients with ES-SCLC [7]. Median overall survival (OS) was 12.3 months for atezolizumab versus 10.3 months for placebo, and median PFS was 5.2 months for atezolizumab versus 4.3 months for placebo [7]. The combination of atezolizumab and chemotherapy significantly prolongs OS and PFS in the first-line treatment of ES-SCLC.

Currently used predictive biomarkers for immune checkpoint inhibitors include PD-L1 expression (on cancer or immune cells), high tumor mutational burden (TMB-H) and/or microsatellite instability (MSI-H) [8,9]. However, none of these biomarkers is robust enough, as only ∼20–30% of cancer patients respond to currently available ICI [10]. In SCLC, none of these biomarkers has been shown to reliably predict a response to atezolizumab [11].

Herein, we present the case of a 57-year-old patient with ES-SCLC exhibiting a long-term, complete response to first-line therapy with atezolizumab, carboplatin, and etoposide. Comprehensive genomic profiling (CGP) was utilized using next-generation sequencing (NGS) technology that revealed potentially underpinning molecular alterations.

## 2 Clinical presentation

**Fig. 1:**
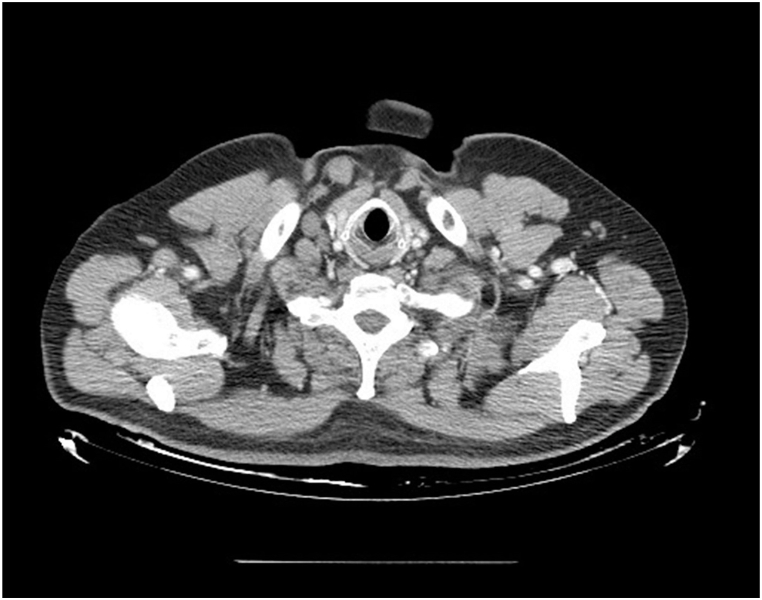
A CT scan of the neck revealed metastasis in the right lymph node of the neck level IV and the right lymph node of the neck level VB.

**Fig. 2:**
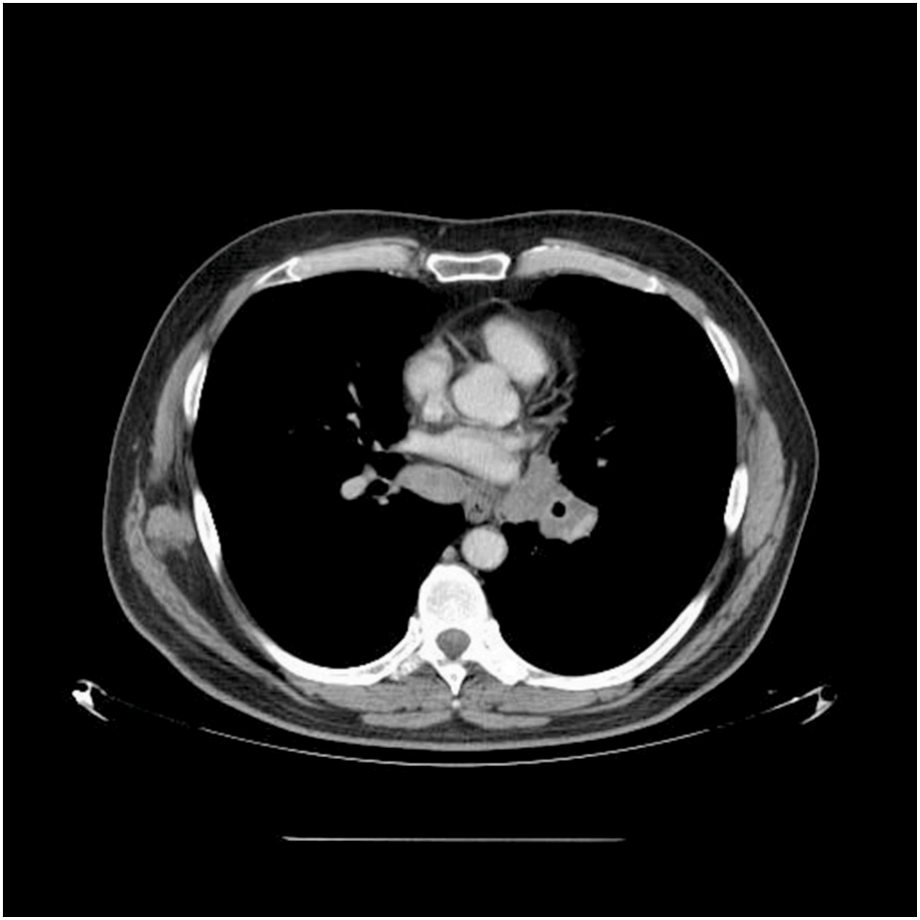
A chest CT scan showed a left hilar tumor with metastases in the mediastinal lymph nodes.

A 57-year-old male smoker presented in December 2018 with a recent history of shortness of breath. He also had a history of type 2 diabetes mellitus, adenoma of the left adrenal gland and shrapnel in his right lung due to the wound in the war. A multislice computed tomography (CT) of the neck showed enlarged right neck lymph nodes (levels IV and VB), highly suggestive of metastatic disease (Fig. 1). Chest CT revealed a 55 mm left hilar mass and enlarged multiple mediastinal lymph nodes. Several micronodules measuring up to 9 mm in both lungs indicated intrapulmonary spread (Fig. 2). Abdominal CT showed a 10 mm hypervascular area in the liver at the border of the VIII and IV segments, radiologically corresponding to either a cavernous hemangioma or a solitary metastasis. Brain CT and skeletal scintigraphy were normal. Magnetic resonance imaging (MRI) of the brain could not be performed because of the shrapnel in the body. A fine needle aspiration cytology of the suspected right cervical lymph node revealed metastatic cancer. A subsequent bronchoscopy and transbronchial biopsy confirmed SCLC. The tumor cells were immunohistochemically positive for TTF-1 and neuroendocrine marker CD56. The patient was diagnosed with ES-SCLC as defined by the Veterans Administration Lung Study Group.

**Fig. 3:**
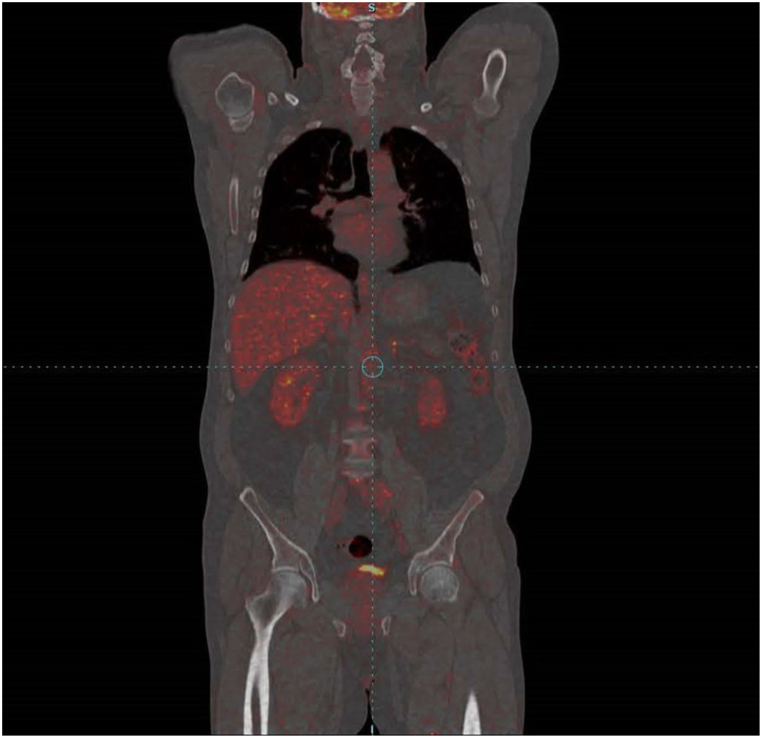
Positron emission tomography/computed tomography (PET/CT) revealed a complete metabolic response without signs of metabolically active disease.

The multidisciplinary team decided to treat with chemotherapy plus immunotherapy with atezolizumab, provided through a donation program. The treatment started in March 2019. Four cycles of chemotherapy with carboplatin (AUC 5, IV day 1) and etoposide (100 mg/m^2^, IV days 1–3) concurrently with atezolizumab (1200 mg, IV day 1) were started. Side effects during the treatment included prolonged grade 2 neutropenia (neutrophil count of 1200/μL in the blood) and a metallic taste in the mouth. The dose of carboplatin was reduced by 25% due to neutropenia. After three cycles of chemoimmunotherapy, a control radiological imaging revealed a significant regression of the mediastinal lymph nodes, while the primary tumor almost completely regressed. On the CT of the abdomen, the suspicious lesion in the liver was stationary. Control US of the neck showed complete regression of right cervical lymph node metastasis. Prophylactic cranial irradiation was not performed after the completion of chemoimmunotherapy. After four cycles of chemoimmunotherapy, maintenance therapy of 28 cycles of atezolizumab (1200 mg, IV day 1) was continued from July 2019 to February 2021. During the maintenance therapy with atezolizumab, side effects were mild skin rash, fatigue and hyperglycemia (blood glucose levels up to 15.1 mmol/L) and insulin therapy was added to oral hypoglycemic agents. On the control CT of the thorax in July 2019, the mediastinal and left hilar lymph nodes were in further regression. After that, radiological imaging was stable. One month after the completion of maintenance therapy with atezolizumab in March 2021, a PET/CT scan was performed, revealing no metabolically active disease (Fig. 3). A close clinical follow-up was recommended, with the last check-up in early December 2022 without recurrent or metastatic disease.

## 3 Comprehensive genomic profiling (CGP)

Retrospective CGP was performed on the primary tumor sample to investigate tumor genomic characteristics, which could explain the success of the therapy. CGP was utilized using the NGS-based FoundationOne CDx assay (Foundation Medicine®, Boston, MA). The assay explores 324 genes in solid tumors for >20 approved targeted therapies [12]. Before CGP, two board-certified pathologists (F.S. and S·V.) reviewed the slides from the primary biopsy sample to confirm the diagnosis and assess the quality of the sample for CGP.

CGP revealed a TMB of 13 mutations/Mb, while the tumor was microsatellite stable (MSI-S). The tumor cells harbored mutations of *RB1* L518fs*6, *ERCC4* R799W and *TP53* C124* genes, consistent with alterations in small cell carcinomas. No other pathogenic genomic alterations were reported.

PD-L1 testing by immunohistochemistry was not performed due to the sparse remaining tissue in the paraffin block.

## 4 Discussion

Our study reports a patient with ES-SCLC achieving a complete and long-lasting response to immunotherapy with atezolizumab combined with chemotherapy. A complete response of patients with ES-SCLC to combined chemoimmunotherapy is unusual and has been reported in approximately 2–2.5% of patients [7,13]. The synergistic effects of the immune- and chemotherapy in our patient could play an important role in activating the immune system against cancer. Indeed, the essential role of immunotherapy concurrent with chemotherapy was demonstrated by the recent approvals of atezolizumab and durvalumab in the first-line treatment of ES-SCLC [5,6]. Regardless of the modest benefit on OS, the effectiveness of atezolizumab in combination with carboplatin and etoposide compared with chemotherapy was also demonstrated in the updated analysis of the IMpower 133 study. After the median follow-up of 22.9 months, OS was 12.3 and 10.3 months (hazard ratio (HR), 0.76). At 18 months, survival was 34.0% and 21.0%, respectively [14]. Real-world clinical practice studies also confirmed the benefit of atezolizumab and chemotherapy in the first-line treatment of ES-SCLC [15]. Based on the phase 3 CASPIAN RCT results, durvalumab was approved in the first-line treatment of ES-SCLC [13]. In the CASPIAN study, untreated patients with ES-SCLC were divided into three groups: platinum with etoposide × six cycles; chemotherapy plus durvalumab × four cycles followed by maintenance durvalumab or chemotherapy plus durvalumab and tremelimumab × four cycles followed by maintenance durvalumab. The combination of durvalumab plus chemotherapy demonstrated improved OS compared with chemotherapy alone (13 vs. 10.3 months, HR, 0.73), and after follow-up, 3-year survival was 17.6% and 5.8%, respectively [16]. In RCT phase 3 KEYNOTE 604, pembrolizumab in combination with chemotherapy and followed by maintenance pembrolizumab compared to chemotherapy showed significant PFS but no significance threshold for OS [17].

Several predictive biomarkers of response to ICI have been validated across cancers, including TMB [18]. A linear relationship between TMB-H and response to ICI has also been described [19]. However, no universal consensus in the definition of TMB-H exists; FoundationOne, the assay we used, defined TMB-H tumors when ≥20 mutations/Mb are present, while the FDA used the threshold of 10 mutations/Mb when approving pembrolizumab in a tumor-agnostic fashion. Nevertheless, moderate/TMB-H (13 mutations/Mb) may partially explain our patient's exceptional response to atezolizumab.

Zhou et al. also confirmed a more favorable outcome for SCLC patients with TMB-H cancers [20]. A retrospective study of patients with advanced solid tumors treated with pembrolizumab revealed that TMB-H tumors (defined as TMB ≥10 mutations/Mb) had a better objective response rate than tumors with low TMB (29% vs. 6%) [21]. Similar findings were reported in SCLC patients whose cancer had TMB-H and were treated with ICI [22,23]. A retrospective study of patients treated with *anti*-PD-1 monotherapy or combined *anti*-PD-1/CTLA-4 inhibitors for relapsed/refractory SCLC revealed a benefit of TMB-H compared with low TMB (median PFS 3.3 months vs. 1.2 months; HR: 0.37; median OS 10.4 months vs. 2.5 months; HR 0.38) [23]. However, these results are inconsistent with the IMpower133 update analysis, which reported a therapeutic benefit of atezolizumab regardless of TMB status [14].

Our CGP revealed *TP53* and *RB1* gene mutations, SCLC's most common genomic alterations [24]. In contrast to their prognostic, the predictive roles of *TP53* and *RB1* mutations in SCLC remain unknown. The case also harbored an *ERCC4* gene mutation, a structure-specific nuclease-encoding gene, whose mutations disrupt the DNA repair mechanisms (nucleotide excision repair/NER gene) [25,26]. In contrast to NSCLC, particularly adenocarcinoma, *ERCC4* mutations are rarely described in SCLC [24,27]. Experimental data indicate that *ERCC4* mutations could be a predictive biomarker of response to platinum-based chemotherapy. Ceccaldi et al. described a case of advanced high-grade serous ovarian carcinoma with *ERCC4* mutation, exhibiting a durable response to first-line platinum therapy and a disease-free interval of 25 months after the diagnosis [28]. Lastly, previous data indicate that SCLC patients with oligometastatic disease (seen in our case) have a better outcome than SCLC patients with polymetastases [29]. All these characteristics probably contributed to our patient's exceptional and long-lasting response to combined chemoimmunotherapy.

## 5 Conclusions

• A complete response to chemoimmunotherapy may be achieved in the case of ES-SCLC.
• Our case study reaffirms the potential values of clinical staging and predictive (NGS) testing in decision-making for treating highly aggressive cancers, such as SCLC.

## Author contributions

All authors made substantial contributions to the conception and design, acquisition of data, or analysis and interpretation of data; participated in drafting the article or revising it critically for important intellectual content; agreed to submit to the current journal; gave final approval of the version to be published; and agree to be accountable for all aspects of the present work.

## Ethical statement

The study was approved by the institutional review board of the University Clinical Hospital Mostar (number of approval: 1217/2022, September 29, 2022). The patient also signed the informed consent to comprehensive genomic profiling and publishing of the case.

## Declaration of competing interest

The authors declare no conflict of interest.
